# Hormone, Targeted, and Combinational Therapies for Breast Cancers: From Humans to Dogs

**DOI:** 10.3390/ijms25020732

**Published:** 2024-01-05

**Authors:** Chiao-Hsu Ke, Chao-Nan Lin, Chen-Si Lin

**Affiliations:** 1 Sustainable Swine Research Center, National Pingtung University of Science and Technology, Pingtung 91201, Taiwan; f08629002@ntu.edu.tw (C.-H.K.); cnlin6@mail.npust.edu.tw (C.-N.L.); 2 Animal Disease Diagnostic Center, College of Veterinary Medicine, National Pingtung University of Science and Technology, Pingtung 91201, Taiwan; 3 Department of Veterinary Medicine, College of Veterinary Medicine, National Pingtung University of Science and Technology, Pingtung 91201, Taiwan; 4 Department of Veterinary Medicine, School of Veterinary Medicine, National Taiwan University, Taipei 10617, Taiwan

**Keywords:** hormone therapy, targeted therapy, comparative oncology, canine mammary gland tumor

## Abstract

Breast cancer (BC) is the most frequent cancer in women. In female dogs, canine mammary gland tumor (CMT) is also the leading neoplasm. Comparative oncology indicates similar tumor behaviors between human BCs (HBCs) and CMTs. Therefore, this review summarizes the current research in hormone and targeted therapies and describes the future prospects for HBCs and CMTs. For hormone receptor-expressing BCs, the first medical intervention is hormone therapy. Monoclonal antibodies against Her2 are proposed for the treatment of Her2+ BCs. However, the major obstacle in hormone therapy or monoclonal antibodies is drug resistance. Therefore, increasing alternatives have been developed to overcome these difficulties. We systemically reviewed publications that reported inhibitors targeting certain molecules in BC cells. The various treatment choices for humans decrease mortality in females with BC. However, the development of hormone or targeted therapies in veterinary medicine is still limited. Even though some clinical trials have been proposed, severe side effects and insufficient case numbers might restrict further explorations. This difficulty highlights the urgent need to develop updated hormone/targeted therapy or novel immunotherapies. Therefore, exploring new therapies to provide more precise use in dogs with CMTs will be the focus of future research. Furthermore, due to the similarities shared by humans and dogs, well-planned prospective clinical trials on the use of combinational or novel immunotherapies in dogs with CMTs to obtain solid results for both humans and dogs can be reasonably anticipated in the future.

## 1. Introduction

**Figure 1:**
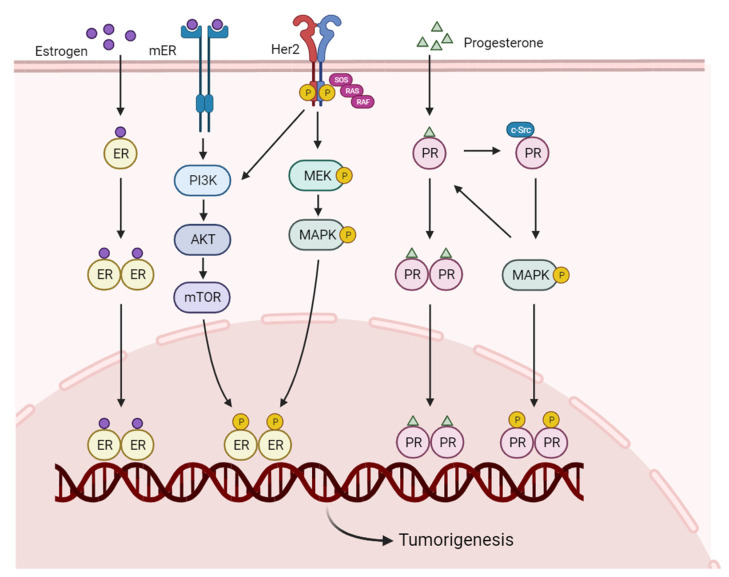
The estrogen receptor, progesterone receptor, and Her2 pathways in breast cancer cells. Two steroid hormones, estrogen and progesterone, directly penetrate through the cell membrane and then bind to the corresponding receptor monomer, which is then dimerized and transported into the nucleus to activate signaling. The extracellular estrogens bind to the membrane receptors and activate the PI3K/AKT signaling. Her2 promotes cell survival and proliferation by activating MAPK and PI3K/AKT pathways. Activation of RAS kinase triggers the activation of the MAPK signaling cascade, which includes the phosphorylation of RAF, MEK, and MAPK. Upon phosphorylation by MEK, MAPK translocates into the nucleus, where it regulates the transcription of genes involved in cell proliferation. Furthermore, progesterone-induced activation of c-Src also provides a hormone-induced MAPK phosphorylation signaling, thus modulating tumorigenesis. c-Src, proto-oncogene tyrosine-protein kinase Src; ER, estrogen receptor; Her2, human epidermal growth factor receptor2; mER, membrane estrogen receptor; mTOR, mammalian target of rapamycin; MAPK, mitogen-activated protein kinase; MEK, MAPK kinase; PI3K, phosphatidylinositol-3-kinase; PR, progesterone receptor; RAF, Rapidly Accelerated Fibrosarcoma; RAS, Rat sarcoma protein; SOS, Son of sevenless.

In 2022, female breast cancer (BC) was the most commonly diagnosed cancer in the United States, with an estimated 287,850 new cases (31%). BC is the second most common cause of cancer-related deaths among females, corresponding to an estimated 61,360 deaths [1]. In breast tissues, the hormones estrogen and progesterone are the primary regulators for proliferation and differentiation [2]. They exert cellular functions and activate specific nuclear receptors, namely, estrogen receptors (ERs) and progesterone receptors (PRs) [3]. Once activated, nuclear receptors directly regulate the transcription of genes that promote cell proliferation and development [4]. BCs are heterogeneous and can be divided into several subtypes according to their molecular and histological features [5]. At the molecular level, five subtypes have been identified: luminal A (ER/PR+, Her2−), luminal B (ER/PR+, Her2+), human epidermal receptor 2 (Her2)-enriched (ER/PR−, Her2+), triple-negative (ER/PR−, Her2−), and normal-like (ER/PR+, Her2−, Ki67−) [6]. These classifications provide important clues for treatment decision making [7]. At present, the hormone receptors (HRs) ER and/or PR are expressed in about 70% of all BC patients. Therefore, hormonotherapy is a major therapeutic strategy [8]. The most frequent medicines are estrogen blockers and aromatase inhibitors (AIs), which prevent hormone production from the ovaries [9]. Furthermore, in the case of BC expressing Her2, several antibodies targeting tumors have been reported. These antibodies and/or analogs bind to different sites of Her2 and disturb the proliferative signaling pathways in cancer cells [10] (Figure 1).

In recent years, comparative oncology has been widely proposed. Canine cancers share several biological and pathological similarities with their human counterparts [11]. In addition to the spontaneous tumor initiation, the clinical similarities between human and canine mammary tumors (CMTs) include hormonal etiology, the molecular characteristics of steroid receptors, and the courses of the diseases [12]. In canines, as in humans, hormones (estrogen and progesterone) moderate the growth and development of mammary glands and participate in carcinogenesis [13]. Approximately 50% of CMTs are malignant [14,15,16], but recent studies have shown an increasing trend in malignant tumors [17]. The prevalence varies by geographic location, and in countries where ovariectomy is not routinely performed, it is more prevalent. In these countries, the prevalence of CMTs in female dogs is three-times higher than that in women [18]. Although clinical staging and histological grading might help standardize treatment protocols, no precision therapies are available in dogs with CMTs. Therefore, over 40% of dogs with CMTs die within one year of diagnosis [19]. This difficulty highlights the urgent need to develop updated therapeutic protocols and targeted therapies.

Though BC is still the most frequently diagnosed type of tumor, BC mortality rates have gradually decreased in recent years. This decline indicates the progress of advances in anti-cancer therapies [20]. Many studies have found highly similar tumor behaviors in HBCs and CMTs. This review summarizes the current hormone, target, and combinational therapies in HBCs. The comparative oncology and published clinical and basic data on established anti-tumor therapies in CMTs are also described here.

## 2. Hormone Therapies

### 2.1. Selective Estrogen Receptor Modulators

**Figure 2:**
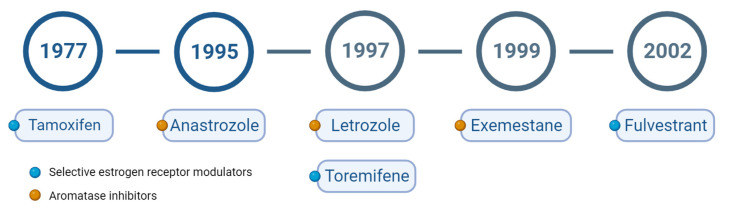
FDA-approved hormone therapies for breast cancers.

Numerous hormone-related drugs were approved from 1977 to recent years (Figure 2). Among these agents, tamoxifen has solid evidence that supports its usefulness as a hormone therapy in treating early breast cancers [21] and metastatic BC in postmenopausal females [22]. The relatively low toxicity of tamoxifen has led to multiple clinical trials. The first single-arm phase 2 trial of tamoxifen was initiated in 1969, in which 22% of recruited patients responded to treatment [23]. Tamoxifen was then compared with other medical interventions in several clinical trials. Although no significant differences in survival were found compared with other agents or medicines, patients with tamoxifen treatment reported less severe side effects [24]. Several clinical trials produced similar findings. One clinical trial evaluated patients with receptor-positive BC, who were randomized to five years of tamoxifen or placebo after surgery. In females treated with tamoxifen, the disease-free survival (DFS) rate was significantly higher than that of the placebo group [25]. In a clinical trial, 1285 patients with BC underwent tumorectomy with axillary lymph node clearance and were treated with tamoxifen for two years. That study demonstrated that a two-year course of tamoxifen therapy was highly associated with a reduction in recurrence and an improvement in overall survival time [26]. Randomized trials of tamoxifen versus other selective estrogen receptor modulators (SERMs) were conducted in hopes of providing greater clinical efficacies than those of tamoxifen. Unfortunately, these agents, idocifene and toremifene, turned out to be equally or less effective than tamoxifen in treating advanced BC [27,28]. However, persistent therapy has been debated, especially if the duration exceeds five years. Therefore, tamoxifen remains the leading SERM, and interest in developing analogs with better anti-tumor activities and reduced toxicity continues [29].

### 2.2. Aromatase Inhibitors

In postmenopausal females, estrogen is poorly produced by ovarian tissues and is predominantly synthesized from nongranular sources by the aromatase enzyme. Aromatase is distributed in several tissues, including subcutaneous fat, liver, muscle, and breast cancer cells [30]. Therefore, inhibition of aromatase has been proposed as a treatment strategy for breast cancer.

The first two generations of aromatase inhibitors (AIs) were effective but simultaneously caused severe side effects because they also inhibited steroid hormones, such as cortisol and aldosterone. Due to the increased specificity of third-generation AIs, the selective inhibition of aromatase did not interfere with the other steroid biosynthesis. These novel AIs, anastrozole, letrozole, and exemestane, have comparable efficacy and have significantly better activities than tamoxifen [31]. These qualities led to the introduction of the first trial that randomized postmenopausal females to tamoxifen or anastrozole. The results showed that anastrozole provided a superior DFS [32] and overall survival time (OST) to those of tamoxifen [33]. In a head-to-head clinical trial, the well-tolerated efficacy of letrozole was compared with that of tamoxifen. This phase 3, double-blind trial recruited 8010 patients and compared their 5-year survival rates. The results showed that the 5-year survival rate was higher in the letrozole group than in the tamoxifen group. Furthermore, some side effects, including thromboembolism, endometrial cancer, and vaginal bleeding, were more common in the tamoxifen group [34]. Therefore, AIs are well-tolerated drugs with manageable side effects in general. The common side effects include musculoskeletal symptoms, osteopenia, osteoporosis, and a heightened risk of fracture [35]. However, the fracture risk appears to increase when patients are undergoing active treatment. The differences in fracture risk between cases with and without AIs were not significantly different after the treatment was completed [36]. Furthermore, because of the lack of partial estrogenic effects, AIs might not enhance the risks of endometrial cancers and thromboembolisms, as tamoxifen does [35,36]. Based on the clinical relevance of anastrozole and letrozole, the FDA approved the use of these two AIs for the initial therapy of hormone-sensitive breast cancer. A clinical trial that compared the efficacy and safety of letrozole and anastrozole was reported. That study included 4136 postmenopausal patients and found that letrozole and anastrozole had similar efficacies [37].

Although AI therapies have met with success in BC in past decades, patients with tamoxifen therapy usually experienced recurrence because of drug resistance and developed side effects. The focus then shifted to combinational therapy, and AIs have been regarded as sequential therapy after tamoxifen treatment [38,39]. The clinical results suggest that postmenopausal patients benefit from a 2-year course of tamoxifen followed by a 3-year course of anastrozole. A decrease in risk for recurrence was noted in patients receiving tamoxifen and anastrozole compared with those receiving tamoxifen only [38]. This result suggested that the sequential use of AIs and tamoxifen provided clinical benefits in BC patients (Table 1).

### 2.3. Resistance to Hormone Therapies

Several molecular mechanisms have been proposed for hormone therapy resistance. Pathways that lead to estrogen-independent sustained activation of estrogen receptors render patients resistant to these hormone drugs: the acquired mutation of *ESR1*, upregulation of miRNAs, and autophagy. The wild-type *ESR1* is bound by an estrogen ligand to enable coactivator recruitment, whereas mutated *ESR1* (*ESR1-MUT*) is constitutively active and unaffected by the AI depletion of estrogen [40,41]. Therefore, in the absence of a ligand, *ESR1-MUT* has increased stability in the active conformation, increased binding to coactivators, and decreased proteolytic degradation [42,43]. The upregulation of miRNAs can be associated with anti-hormone therapies through the activation of alternate pathways or the inhibition of ER expression. MiR-155 stimulated the activation of STAT3 signaling pathways, which is associated with cell survival, thus leading to resistance to tamoxifen [44,45]. MiR-221 and miR-222 regulated several oncogenic pathways, such as Wnt, and their high expression was found to be correlated with tamoxifen resistance [46,47]. In breast cancer cells, autophagy allows cells to survive under endoplasmic reticulum stress, which increases the drug resistance following anti-hormone therapies [48]. For example, the mammalian target of rapamycin (mTOR) kinase is the major component of two mTOR complexes, mTORC1 and mTORC2. mTORC1 is a major downstream target of the PI3K/AKT pathway and a negative regulator of autophagy. Therefore, the downregulation of mTORC1 by PI3K/AKT signaling inhibition results in increased autophagy, which facilitates the resistance to tamoxifen in breast cancer cells [49]. Abnormal interactions between the cyclin D-CDK 4/6-retinoblasoma (Rb) pathways, aberrant modification by histone deacetylases (HDACs), and dysregulation of the cellular microenvironment and immune responses also cause resistance to hormone therapy in breast cancer [50]. Thus, these studies on therapy resistance trigger the development of new drugs/agents targeting these pathways. Although the newly developed drugs fail to reverse drug resistance, they are expected to reinstate sensitivity to endocrine therapy through the inhibition of other pathways or targets.

## 3. Targeted Therapies

**Figure 3:**
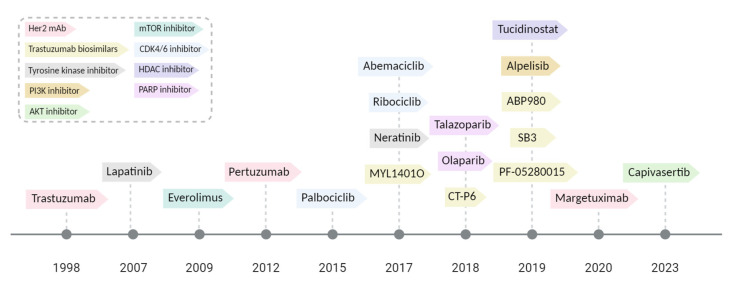
Timeline of FDA-approved targeted therapies for anti-breast cancer agents.

In recent years, targeted therapies, including Her-2-targeted and small molecular agents, have gradually been approved by the FDA in the treatment of BC (Figure 3). A summary of the included studies is shown in Table 2.

### 3.1. Her2-Targted Therapies

#### 3.1.1. Monoclonal Antibodies

Human epidermal growth factor receptor-2 (Her2) is overexpressed in 15% to 20% of early-stage BCs [55], which makes it an ideal target for medical intervention. A humanized monoclonal antibody against Her2, trastuzumab (Herceptin), was first approved by the FDA for Her2+ BC patients. Trastuzumab binds to the extracellular domain of Her2, suppresses cell cycle arrest, and mediates antibody-dependent cell-mediated cytotoxicity [56]. Because of its specificity to Her2, trastuzumab provides remarkable success in treating Her2+ BC patients. Clinical studies have shown that a combination of trastuzumab and chemotherapy produces more favorable outcomes than chemotherapy alone [57,58]. Therefore, trastuzumab was recommended as the treatment choice for patients with Her2+ BC, which revolutionized the outcomes for patients suffering from this disease. However, although trastuzumab provides improved outcomes, some patients develop drug resistance and disease relapse [59].

Resistant mechanisms to trastuzumab encompass various factors, including steric effects, alternative elevations of different tyrosine kinase receptors, and intracellular alterations. Steric effects refer to structural mutations in the Her2 protein, resulting in the inhibition of trastuzumab binding. This leads to the overexpression of other tyrosine kinase receptors, thereby evading the Her2 signaling pathway blockage [60]. In other words, during this mutation, a truncated isoform with constitutive kinase activity emerges, bypassing trastuzumab’s neutralizing effects [61]. Notably, trastuzumab fails to prevent Her3 dimerization, potentially leading to trastuzumab-mediated inhibitory signaling pathways being compromised by the overexpression of Her3 [62]. Suppression of the Her2 signaling pathway by trastuzumab prompts cancer cells to compensate through increased reliance on Her3 signaling [63]. Last, understudied mechanisms contributing to trastuzumab resistance involve intracellular alterations. Research indicates that patients with *PTEN* loss exhibit significantly lower overall responses to trastuzumab compared to those with wild-type *PTEN* [64]. Clinical findings suggest that trastuzumab-treated patients lacking *PIK3CA*-activating mutations experience improved PFS compared to their mutated counterparts [65]. In vitro studies further demonstrate increased trastuzumab resistance in Her2+ breast cancer cells with *PIK3CA*-activating mutations [66]. These results highlight the role of the constitutive activation of the PI3K/AKT pathway, stemming from *PTEN* deficiency or *PIK3CA*-activating mutations, in contributing to trastuzumab resistance. While the detailed mechanisms necessitate further elucidation, the emergence of trastuzumab resistance and disease relapse in several breast cancer patients underscores the urgency for the development of novel medical interventions.

Previous studies suggested that antibodies targeting multiple domains in Her2 could potentially elicit synergistic anti-neoplastic effects [67]. Therefore, the development of the second-generation humanized monoclonal antibody, pertuzumab, was proposed. Pertuzumab binds to Her1, Her3, and Her4, effectively inhibiting downstream tumor signaling pathways [68]. Recognizing the synergistic effects demonstrated by trastuzumab and pertuzumab, the idea of combining these two monoclonal antibodies with chemotherapy gained traction, leading to the initiation of clinical trials. In a clinical trial, patients with Her2+ BC were randomized into two groups: one receiving pertuzumab, trastuzumab, and docetaxel (pertuzumab combination), and the other receiving trastuzumab and docetaxel (placebo combination). The results from this trial revealed a significant improvement in median overall survival, extending to 56.5 months with the addition of pertuzumab, highlighting the promising efficacy of this combined therapeutic approach [69]. Another phase 2 clinical study involving women with Her2+ BC corroborated these findings, showing that patients administered pertuzumab, trastuzumab, and docetaxel exhibited a significantly enhanced treatment response rate compared to those receiving trastuzumab and docetaxel alone [70]. These clinical trials collectively underscore the enhanced survival and treatment responses achieved through pertuzumab and trastuzumab combinational therapies.

In light of these discoveries, the field of developmental therapeutic antibodies has witnessed a surge in momentum, propelling the clinical advancement of novel HER2-targeted monoclonal antibodies. Margetuximab is a chimeric anti-Her2 monoclonal antibody engineered with an Fc domain modification to enhance binding to FcγRIIIa and reduce affinity for its inhibitory counterpart, FcγRIIB [71]. Antibody-dependent cytotoxicity assays have demonstrated the heightened activity of margetuximab against Her2+ cancer cells compared to trastuzumab surrogates in donors possessing a low-affinity variant of FcγRIIIa [71]. A phase 1 clinical trial recruited patients with metastatic BC. Notably, 11 out of 28 BC patients exhibited tumor reduction, all of whom had previously received at least one Her2-targeted therapy [72]. The clinical efficacy of margetuximab is currently under scrutiny in a phase 3 trial, comparing margetuximab combined with chemotherapy against trastuzumab plus chemotherapy. This trial involves patients with Her2+ metastatic BC who have experienced progression following Her2-targeted therapy. Preliminary results from 536 patients with Her2+ metastatic BC revealed that margetuximab led to an improvement in PFS compared to trastuzumab (median of 6 months vs. 5 months) [73].

#### 3.1.2. Trastuzumab Biosimilars

Clinical trials have demonstrated the ideal efficacy and safety of trastuzumab; however, over 90% of patients in several low-income countries had no access to trastuzumab owing to economic restraints [74]. A lower-cost alternative, trastuzumab biosimilars, has the potential to increase accessibility in the treatment of Her2+ BC. Biosimilars are biological agents that are created from living cells, contain versions of the reference products’ substances, and have similar pharmacokinetic and pharmacodynamic properties to the original products [10]. Although they may have minor differences from their parental components, biosimilars usually share similar biological characteristics, efficacy, and safety [75]. Several trastuzumab biosimilars have undergone phase 3 trials in patients with early and metastatic BC [76,77,78]. A multicenter phase 3 randomized clinical trial assessed the efficacy of trastuzumab and a trastuzumab biosimilar (HD201) in patients with Her2+ BC. HD201 demonstrated equivalence to the referent trastuzumab in terms of efficacy in the response rate and a similar safety profile [76]. Similar results were proposed in a clinical trial recruiting patients with Her2+ metastatic BC. A proposed trastuzumab biosimilar resulted in an equivalent overall response rate compared with trastuzumab [77]. A phase 3 randomized clinical trial included women with Her2+ early BC and evaluated the efficacy and safety between trastuzumab and a trastuzumab biosimilar (ABP980). The results showed that the trastuzumab biosimilar (ABP980) shared high similar efficacy and safety with trastuzumab [78]. Based on that success, five trastuzumab biosimilars have been approved by the US FDA, with additional candidates still underdeveloped. Trastuzumab-dkst (MYL1401O) was the first to receive FDA approval in 2017, followed by tastuzumab-pkrb (CT-P6) in 2018 and trastuzumab-dttb (SB3), trastuzumab-qyyp (PF-05280015), and trastuzumab-anns (ABP980) in 2019 [10]. After FDA approval, one study evaluated the effectiveness of MYL1401O in patients with early Her2+ BC. That study demonstrated similar response rates in patients receiving MYL1401O plus chemotherapy or trastuzumab plus chemotherapy [79]. Taken together, the clinical relevance of trastuzumab biosimilars has been revealed, showing its value as an excellent cost-effective alternative to trastuzumab.

The progress of biosimilars presents significant potential for improving access to biological therapies and curbing healthcare expenses. However, tackling these challenges is pivotal for promoting a more well-rounded exploration of biosimilars. Thus, a comprehensive strategy is essential to surmount economic constraints in the biosimilars and antibody landscapes. Collaborative efforts involving regulators, industry stakeholders, and healthcare professionals might pave the way for a more sustainable exploration of biosimilars.

#### 3.1.3. Tyrosine Kinase Inhibitor

Lapatinib is a reversible inhibitor of EGFR (Her1) and Her2 [80,81]. Therefore, trastuzumab and lapatinib have complementary mechanisms of action and synergistic anti-tumor activity in Her2+ BC. A phase 3 clinical trial (NeoALTTO) recruited patients with Her2+ tumors greater than 2 cm in diameter. Significantly higher response rates were found in patients receiving trastuzumab plus lapatinib than in those with trastuzumab alone [82]. An extended study reported, however, that overall survival and PFS were similar between these groups [83]. In a phase 2 EORTC trial, Her2+ BC patients benefited from trastuzumab and lapatinib therapy with an increased response rate. However, PFS did not increase compared with the control arm [84]. Although lapatinib plus trastuzumab increased pathological complete response and improved some outcomes for Her2+ BC patients, these therapies seem to have mild impacts on extended overall survival. These findings might result from trastuzumab resistance, but lapatinib is reported to overcome trastuzumab resistance in patients [80,81]. Therefore, the clinical utility of lapatinib in combination with other drugs for trastuzumab-resistant BC has been proposed [85]. A clinical trial demonstrated that women with Her2+-advanced BC benefited from lapatinib plus capecitabine, which significantly increased PFS. The median PFS was 8.4 months in the combination therapy group, as compared with 4.4 months in the control arm [86]. The efficacy and safety of lapatinib plus letrozole were well tolerated. Postmenopausal women with HR+/HER2+ BC were randomized to treatment with lapatinib plus letrozole versus letrozole plus placebo. The addition of lapatinib to letrozole was well tolerated and resulted in a significantly increased PFS and overall response rate [87,88]. Taken together, lapatinib in combination with capecitabine was superior to capecitabine alone, and the lapatinib–letrozole combination was more efficacious than letrozole alone [10]. Thus, these combinations have been approved by the FDA for BC treatment.

In contrast to lapatinib, neratinib is an irreversible pan-HER TKI that targets Her1, Her2, and Her4 [89]. A phase 1/2 clinical study assessed the dose-escalation, safety, efficacy, and pharmacokinetics in patients with Her2+ BC. Though a high response rate was found (73%) in patients receiving neratinib plus paclitaxel, a high toxicity rate was also identified, suggesting the need for a dose reduction [90]. A multicenter, randomized, phase 3 trial (ExteNET) was then conducted to assess the benefits and risks of neratinib. Patients who had Her2+ early-stage BC and had experienced trastuzumab therapy were recruited and randomly assigned to receive neratinib or placebo. This trial demonstrated that neratinib for 12 months significantly improved 2-year invasive PFS when given to women with Her2-positive breast cancer after trastuzumab therapy [91].

In clinical practice, lapatinib and neratinib are the only TKIs approved by the FDA for Her2+ BC patients. Several TKI candidates, such as pyrotinib (approved by the Chinse regulatory authority) and tucatinib, are still under investigation. Whether treatment with a single multi-kinase inhibitor or combined treatment with other anti-tumor drugs, TKIs have a high potential in the treatment of trastuzumab-resistant BC patients.

### 3.2. PI3K/AKT/mTOR Pathway Target Inhibitors

#### 3.2.1. PI3K Inhibitors

**Figure 4:**
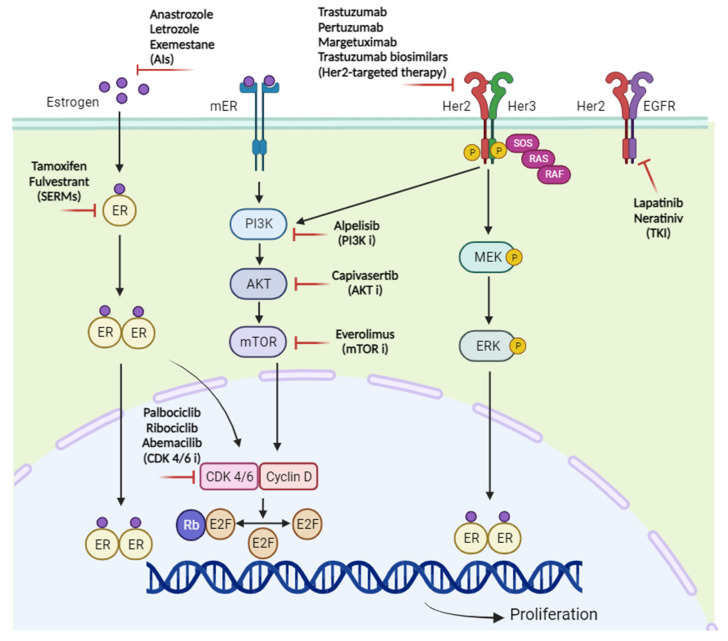
Crosstalk between signaling and pharmacological mechanisms of hormone therapies and targeted agents. AIs, aromatase inhibitors; CDK, cyclin-dependent kinases; EGFR, epidermal growth factor receptor; ER, estrogen receptor; Her2, human epidermal growth factor receptor2; Her3, human epidermal growth factor receptor3; mER, membrane estrogen receptor; mTOR, mammalian target of ra-pamycin; MEK, mitogen-activated protein kinase; PI3K, phosphatidylino-sitol-3-kinase; RAF, Rapidly Accelerated Fibrosarcoma; RAS, Rat sar-coma protein; Rb, retinoblastoma; SERMs, selective estrogen receptor modulators; SOS, Son of sevenless; TKI, tyrosine kinase inhibitor.

Genetic dysregulation in the phosphoinositide 3-kinase (PI3K) pathway, known as mutations of the PI3K catalytic alpha subunit (*PIK3CA*), is usually observed in BC. The first-generation PI3K inhibitors (PI3Ki) are known as pan-inhibitors of PI3K, as they target all four isoforms of class I PI3Ks (α, β, γ, and δ) [92,93]. Because these inhibitors provide widely inhibitory activities, they lead to a high prevalence of side effects, which limit their therapeutic purposes and cause treatment difficulties [94,95,96]. The receptor mechanisms involved in the progression of BC are summarized in Figure 4.

In BC, *PIK3CA*-activating mutations occur in about 40% of cases, and the pan-PI3Ki most commonly used are buparlisib and pictilisib. A phase 3 clinical trial investigated the efficacy and safety of buparlisib plus fulvestrant in HR+/Her2− postmenopausal patients, whose BC was progressive during or after treatment with AIs. These patients were then randomized to receive buparlisib plus fulvestrant or placebo. However, the trial demonstrated relatively mild effects on PFS, and many patients receiving buparlisib treatment withdrew from the trial due to the severe side effects. These adverse effects included higher transaminase levels, hyperglycemia, rash, and mood disorders that influenced the potential benefits of therapy [97]. The abilities of buparlisib plus fulvestrant were then tested in another trial. The results showed that combinational therapy was correlated with improved PFS, especially in those with *PIK3CA* mutations [98]. In the phase 2/3 clinical trial, the combination of buparlisib plus paclitaxel in BC patients as first-line treatment was evaluated. Unfortunately, combinational therapy did not improve the PFS of patients, as compared with paclitaxel alone. Furthermore, buparlisib treatment led to a higher frequency of serious side effects, including diarrhea, rash, hyperglycemia, and nausea [51]. The discrepancy between these trials suggested that the effects and the tolerability of buparlisib still need to be elucidated.

Pictilisib, an orally delivered drug, is a pan-PI3Ki, which shares similarly high efficacy and side effects with those of buparlisib. A randomized, double-blind, phase 2 clinical trial investigated the efficacy and safety of pictilisib plus fulvestrant in HR+/Her2− postmenopausal patients but found no difference in median PFS in the combination treatments. However, patients with pictilisib suffered from drug toxicity, rash, diarrhea, increased transaminase level, hyperglycemia, pneumonitis, broncho-pneumonitis, and fatigue. These severe side effects might limit the treatment effectiveness of pictilisib [99]. Another phase 2 randomized placebo-controlled clinical trial failed to meet the primary endpoint. This study evaluated the benefit of pictilisib plus paclitaxel versus paclitaxel alone in HR+/Her2− BC patients. No significant differences in PFS and overall responses were found, but high toxicity was induced by the pictilisib intervention. Thus, the severe side effects caused by pictilisib resulted in discontinuation of the treatment [100].

Due to the low specificity of pan-PI3Ki, which usually causes considerable adverse effects, PI3K isoform-specific inhibitors were gradually proposed. Alpelisib and taselisib have stronger inhibitory effects and lower incidence of adverse events than pan-PI3Ki. Alpelisib was the first oral inhibitor approved by the US FDA, and it can specifically target the p110α isoform of wild-type PI3Kα [101,102]. A phase 1 trial was primarily carried out. Patients with BC and *PKI3CA* mutations were recruited and demonstrated high sensitivity to a single use of alpelisib. Frequent side effects included hyperglycemia, nausea, rash, diarrhea, fatigue, and mucositis, but a well-tolerated effect was observed. The median PFS was increased by 5.5 months among all the *PIK3*-mutated BC patients [103]. A phase 1b clinical study showed an increased median PFS in patients with *PKI3CA* alterations [104]. These findings triggered the motivation to conduct clinical trials on a larger scale. A phase 3 randomized trial was conducted to assess the safety and efficacy of alpelisib plus fulvestrant versus fulvestrant alone in postmenopausal women with lumina BC. In the *PKI3CA*-mutated group, the median PFS of combination therapy significantly increased compared with that of fulvestrant alone [95,105]. However, alpelisib demonstrated a high incidence of side effects, such as hyperglycemia, diarrhea, nausea, and rash. Due to these issues, 25% of patients in this trial shifted to fulvestrant therapy [106].

Current research and innovative strategies in the field of PI3K inhibitors are actively working towards mitigating adverse effects and enhancing the safety profile of PI3K inhibitors. However, their clinical application has faced challenges due to side effects that may affect patient tolerance and treatment outcomes [107]. One well-studied strategy is isoform-specific inhibition, where studies investigate inhibitors designed to selectively target PI3K isoforms [108]. By homing in on specific isoforms, it is anticipated that the adverse effects associated with non-selective inhibition can be relieved. This approach aims to achieve a more precise therapeutic effect while reducing off-target effects. The exploration of isoform-specific inhibition holds significant promise for improving the safety profile of PI3K inhibitors, thereby advancing their clinical efficacy in cancer therapy. For example, taselisib is a selective PI3Ki; it can selectively inhibit p100α, β, and γ isoforms, thereby exhibiting better selectivity for the mutant PI3Kα isoform [109]. In a phase 1 study, taselisib treatment was associated with markable tumor-suppressing effects in patients with *PIK3CA*-mutated metastatic BC [109]. Based on these encouraging findings, a phase 3 trial was conducted and showed outstanding effects for the combination of taselisib and fulvestrant against *PIK3CA*-mutated BC patients. This trial recruited postmenopausal women and demonstrated that median PFS was significantly increased in *PIK3CA*-mutant patients with combinational therapy. Conversely, with *PIK3CA*-wild-type tumors, no significant difference was found between patients receiving taselisib plus fulvestrant and fulvestrant alone. However, further studies suggested that a high incidence of adverse effects caused by taselisib, including diarrhea and hyperglycemia, resulted in treatment discontinuation [110,111].

Taken together, *PI3K* gene mutations are related to tumor proliferation and metastasis, and *PKI3CA* mutations usually occur in BC patients. The pan-inhibition of PI3K often led to serious side effects instead of satisfactory therapeutic effects. Studies were then shifted to PI3K isoform-specific inhibitors that can specify the target; however, ways to minimize the adverse effects caused by selective PI3Ki will require further investigations.

#### 3.2.2. AKT Inhibitors

AKT is a vital characteristic of the PI3K intracellular pathway; it regulates cell proliferation, survival, and metabolism [112]. Three AKT isoforms, namely, AKT1, AKT2, and AKT3, have been reported. In cancer cells, AKT1 is involved in cell proliferation and growth, thereby facilitating tumor development and suppressing apoptosis. AKT2 participates in cytoskeleton dynamics and, therefore, favors tumor invasion and metastasis. Although the activation of AKT3 in cancer cells remains controversial, it has been speculated to be involved in cell proliferation [113,114].

Hyperactivation of the PI3K/AKT/mTOR pathway is an oncogenic driver in BC and has a correlation with hormone therapy resistance. AKT is altered in about 7% of BC [115], so it represents a potential therapeutic target in patients with BC [116]. The main clinical trial was proposed by FAKTION and investigated the efficacy and safety of an AKT inhibitor, capivasertib. This phase 2 clinical study enrolled postmenopausal women with HR+/Her2− advanced breast cancers who had relapsed or progressed after previous AI treatment. Patients receiving capivasertib plus fulvestrant had improved PFS (10.3 months), as compared with those receiving fulvestrant alone (4.8 months) [117]. Trials that investigated AKT inhibitors with chemotherapy were also reported. A randomized placebo-controlled phase 2 trial (PAKT) evaluated the efficacy of capivasertib plus paclitaxel. The population was stratified by *PIK3CA/AKT1/PTEN* mutational status by next-generation sequencing. In patients with *PIK3CA/AKT1/PTEN* mutation, PFS was significantly extended with capivasertib plus paclitaxel (9.3 months) compared with controls (3.7 months) [52]. Furthermore, in an updated report, these patients showed a favorable trend in OST for capivasertib plus paclitaxel [118].

Therefore, AKT inhibitors represent a promising avenue for cancer therapy, particularly in breast cancer where the PI3K/AKT/mTOR pathway is frequently dysregulated. Clinical trials evaluating AKT inhibitors have shown encouraging results, with capivasertib being a notable example, especially in patients with specific molecular alterations. While AKT inhibitors show promising efficacies in clinics, their development faces certain challenges. One challenge is the potential for off-target effects, as AKT is involved in various cellular processes beyond cancer. Achieving selectivity for cancer cells while minimizing adverse effects on normal cells remains a critical consideration. Second, the complexity of the PI3K/AKT/mTOR signaling pathways involves multiple components and crosstalk with other signaling pathways. Identifying the optimal combination therapies and understanding the context-specific effects of AKT inhibition are areas that require further exploration. In the future, advancements in drug design and technology may lead to the development of more selective and potent AKT inhibitors, minimizing off-target effects and improving overall therapeutic profiles.

#### 3.2.3. mTOR Inhibitors

mTOR is a downstream target of the PI3K/AKT and adenosine monophosphate-activated protein kinase (AMPK) signaling pathways. As a member of these pathways, mTOR is a regulator of cell proliferation and metabolism. The activation of mutations is usually found in the mTOR pathway, including *PIK3CA*, AKT1, and mTOR mutations. Therefore, drugs/agents targeting mTOR have been developed for cancer treatment. The mTOR inhibitor, everolimus, is a targeted drug for HR+/Her2− metastatic BC patients.

In a randomized phase 2 clinical study, patients with ER+ BC were enrolled. The efficacy of everolimus plus letrozole was compared against letrozole alone. The results showed that clinical responses were better in patients receiving everolimus, which increased the letrozole efficacy in the neoadjuvant setting [119]. Furthermore, that study assessed the Ki67 expression in tumors at baseline and after 15 days of medical intervention. In tumors of the everolimus and placebo arms, the Ki67 expression significantly decreased in the everolimus group. This result indicated a greater anti-neoplastic response for everolimus-treated patients. Another phase 2 study evaluated efficacies between everolimus plus tamoxifen and tamoxifen alone. This clinical trial recruited patients with HR+/Her2− advanced BC who had failed AI treatments. The clinical benefit rate of everolimus plus tamoxifen was 61%, as compared with 42% with tamoxifen alone. Improved PFS was found in the combination arm (8.6 months), as compared with the tamoxifen arm (4.5 months) [53]. The phase 3 trial (BOLERO-2) enrolled patients with HR+ advanced BC, who were randomized to receive everolimus combined with exemestane versus exemestane alone. The median PFS was significantly improved in the everolimus plus exemestane arm (6.9 months) compared with the control arm (2.8 months) [120]. An extended report outlined that, at a median follow-up of 18 months, improved PFS was found in patients with the combination therapy, as compared with those who received exemestane alone [121].

### 3.3. CDK4/6 Inhibitors

Cyclin-dependent kinase (CDK) belongs to the serine/threonine protein kinase family and is involved in cell cycle regulation. CDK4 and CDK6 are correlated to D-type cyclins and promote the initiation of progression from the G1 phase to the S phase. Thus, CDK4/6 inhibitors can effectively inhibit the progression of cancer cells from the G1 phase to the S phase. In recent years, CDK inhibitors have been proposed for the management of hormone receptor-positive breast cancers. Three CDK4/6 inhibitors, palbociclib, ribociclib, and abemacicilib, are approved by the US FDA for breast cancers in combination with hormone therapies [122,123,124]. Double suppression of CDK4/6 and ER signals, known as palbociclib with an AI, can control the cell cycle and block the proliferation of breast cancer cells, so it is the first-line choice in postmenopausal females [125].

Palbociclib was the first CDK4/6 inhibitor to be approved by the FDA for breast cancer in combination with AIs in 2015. In phase 2 and 3 clinical trials, postmenopausal patients were given palbociclib plus letrozole or placebo plus letrozole. The results showed that PFS was significantly higher in the palbociclib plus letrozole groups [126,127]. After these trials, a study that assessed the efficacy and safety of palbociclib plus fulvestrant was conducted to evaluate women with advanced BC. Patients with these drugs improved their PFS, with a 58% reduction in progression [128]. Palbociclib is generally well tolerated, with certain side effects. Fatigue, nausea, leukopenia, and neutropenia are commonly reported [122,123,124]. Nevertheless, the overall tolerability was considered to be manageable, which led to a positive quality of life in BC patients [129,130].

In 2017, ribociclib became the second CDK4/6 inhibitor to receive FDA approval for HR+/HER2- advanced or metastatic BC. A clinical trial compared patients receiving ribociclib plus letrozole or letrozole alone. The results were similar to those for palbociclib, in which patients with ribociclib in combination with letrozole had increased PFS [131]. The efficacy and tolerability of the combination were reported in an extended study [132], which also noted that improved PFS and OST were found. In addition to AIs, ribociclib is also being investigated in combination with fulvestrant, and this combinational therapy significantly increased the PFS in BC patients [133]. The results showed that patients benefited from ribociclib plus fulvestrant, showing an increased OST and a decreased relative risk of death [134]. A phase 3 clinical trial evaluated the efficacy and safety of ribociclib as first-line therapy for females with breast cancers. Compared with the placebo group, the groups with ribociclib plus tamoxifen or AIs had significantly higher PFS [135] and OST [136].

Abemaciclib is noted to be the most potent CDK4/6 inhibitor, as it can cross the blood–brain barrier due to its unique structure. This ability may be beneficial for patients with brain metastases, and this medicine has also been approved by the FDA for women with advanced BC. Abemaciclib showed promising clinical activities after 12 months in patients, with an overall response rate of 19.7% and a 6-month increase in PFS [54,122]. Furthermore, patients significantly benefited from the combination of abemaciclib and fulvestrant, with higher PFS [137] and OST [137] compared with those with fulvestrant alone.

For BC treatment, CDK4/6 inhibitors are usually combined with fulvestrant or AIs and have shown well-tolerated efficacy, with manageable side effects. These findings suggested that CDK4/6 inhibitors combined with fulvestrant are a potentially promising choice for the first-line treatment of patients with BC.

### 3.4. HDAC Inhibitors

Histone deacetylase (HDAC) is an enzyme that regulates gene expression and modifies the chromosome structure. Epigenetic alteration modified by HDAC results in aberrant gene expression, leading to disease progression and resistance to hormone therapy in BC patients [138,139]. These modifications/alterations can be reversibly modulated by HDAC inhibitors [140]. Tucidinostat, also known as chidamide, is a selective HDAC inhibitor that suppresses Class I and Class IIb HDACs. A randomized, phase 3 clinical trial enrolled postmenopausal women with HR+/Her2− BC who had relapsed or progressed after at least one hormone therapy. Tucidinostat plus exemestane significantly improved PFS compared with placebo plus exemestane in patients. Though hematological adverse events were more common in the tucidinostat plus exemestane group, these findings provide a new treatment option for patients with relapsed diseases [141].

### 3.5. PARP Inhibitors

DNA damage and deficiencies of repair are vital features of cancer pathology. Normal cells will maintain their genome integrity through DNA damage response (DDR) pathways, which protect them from DNA damage [142]. At least 450 proteins or molecules are involved in DDR pathways, including poly (ADP-ribose) polymerase 1 (PARP1) and PARP2 [143]. Furthermore, PARPs moderate transcription, apoptosis, and immune function [144]. These multiple mechanisms could result in increased PARP inhibitor efficacy.

PARP inhibitors bind to PARP, inhibiting PARylation, and also trap inactivated PARP in DNA, thereby blocking replication forks, which, in turn, leads to collapse and the generation of double-strand breaks in cancer cells [143,145,146]. Two PARP inhibitors, Olaparib and talazoparib, have been approved by the US FDA and European Medicines Agency (EMA) as monotherapy for BC patients. Several PARP inhibitors (niraparib, rucaparib, and veliparib) are also being investigated; for example, veliparib is currently in phase 3 clinical trials for the treatment of HER2- metastatic BC and has showed promising outcomes [147]. A randomized, multicenter, phase 3 clinical trial (OlympiAD) compared the efficacy and safety of olaparib versus single-agent standard therapy of the physician’s choice (TPC: capecitabine, eribulin, or vinorelbine) in patients with Her2- metastatic BC. Median PFS was significantly increased with olaparib (7.0 months) compared with the control arm (4.2 months) [148]. However, in an extended analysis, no significant differences were detected in median overall survival with olaparib (19.3 months) versus controls (17.1 months) [149]. EMBRACA was a randomized, multicenter, phase 3 clinical trial that assessed the efficacy and safety of talazoparib versus single-agent standard TPC. In that study, median PFS was significantly longer in patients with talazoparib treatment (8.6 months) than in those with TPC therapy (5.6 months) [150]. In the final overall survival analysis, however, no significant difference was found in patients receiving talazoparib (19.3 months) or TPC (19.5 months) [151]. Although no significant difference in overall survival was found, olaparib and talazoparib monotherapies have demonstrated significant PFS benefits compared with chemotherapy. Common side effects are manageable through supportive treatment and dose interruptions/reductions. Therefore, the advent of PARP inhibitors has potential benefits for BC treatment.

## 4. Therapies for Triple-Negative Breast Cancer (TNBC)

### 4.1. Immune Checkpoint Inhibitors: Programmed Death-Ligand 1 (PD-L1)

Immune checkpoint inhibition using the antibodies against PD-L1 has shed light on TNBC. Several anti-PD-L1 antibodies, for example, atezolizumab, avelumab, and durvalumab, were investigated and reported to significantly increase OST in TNBC patients. A phase 3 clinical trial evaluated the effects of atezolizumab in combination with nab-paclitaxel. Among the patients with PD-L1-positive tumors, these combinational therapies significantly prolonged the OST (25 months) compared with placebo plus nab-paclitaxel (15.5 months) [152]. A clinical trial recruited 33 patients with metastatic TNBC and examined the safety, tolerability, and clinical activity of atezolizumab. All patients showed at least one treatment-related adverse event, and 73% of patients experienced grade 3 adverse events. Nevertheless, no death was observed, and the median PFS and OST were 5.5 months and 14.7 months, respectively [153].

A phase 1b clinical trial using avelumab reported a higher overall response rate in patients with PD-L1-positive tumors (22.2% versus 2.6%) [154]. Several trials are also conducted with durvalumab for patients with metastatic TNBC. A phase 1b trial reported the therapeutic effects of durvalumab and a peptide vaccine in patients with stage 2 or 3 TNBC. The promising results of PD-L1 therapies in TNBC progressed the utility in TNBC. Furthermore, PD-L1 antibodies alone or in combination with different anti-tumor agents are currently under investigation.

### 4.2. MicroRNA-Based Gene Therapy and Biomimetic Carriers

The ongoing and recent discovery of the regulatory effects of miRNA remains in a preclinical state, in which miRNA can downregulate PD-L1 expression in TNBC cells. Furthermore, the use of miRNA as a therapy for TNBC not only targets the tumor cells but also microenvironmental components. Numerous studies showed that several miRNAs exhibited anti-tumor properties in TNBC cells. For example, miR-195-5p and miR-497-5p can inhibit tumoral PD-L1 expression in MDA-MB-231 cells. MiR-424-50 can inhibit PD-L1 transcript expression in MDA-MB-231 cells and stimulate interferon production in the tumor microenvironment [155]. Taken together, these preliminary studies provide further direction for the utility of miRNA in TNBC patients. The use of miRNAs as therapeutics is a promising new potential modality for the treatment of TNBC.

Carriers that deliver therapeutic agents to target TNBCs were also reported. These techniques may provide the patients with personalized, specific, and safe tumor-associated delivery in the future. Molinaro et al. reported that leukosomes, a kind of biomimetic nanovesicle, can effectively deliver a chemotherapeutic agent to tumors in the syngeneic TNBC murine model [156]. The leukosomes can recruit immune cells within the tumors, thereby decreasing the tumor volume. Kang et al. demonstrated that neutrophil membrane-coated nanoparticles can capture circulating tumor cells and accumulate in metastatic tumors in mice bearing TNBC cells [157].

Some limitations in microRNA-based therapy for breast cancers should be considered. First, the variability in certain microRNAs across different patients might influence the efficacy of the TNBC patients [158]. Individual variation in microRNAs will alter the biogenesis of the microRNA, which makes significant impacts on the transcription, maturation, and target specificity [159]. Therefore, microRNA-based therapy responses vary from patient to patient, and these dissimilarities result from genetic variability [160,161]. Second, the biological functions of microRNA in different types of tumors should be taken into consideration. For example, in breast cancer cells, miR-142-3p stimulated cell apoptosis and cell cycle arrest [162], whereas miR-142-3p increased the cell viability of gastric cancer cells [163]. In summary, although the potential of microRNA-based therapy is fascinating, many more obstacles need to be addressed. Further investigation of microRNA targeting genes and their functions is still needed to elucidate and, thereby, provide precision medicine for patients with TNBC.

### 4.3. Single-Cell Sequencing and Personalized Medicine

Single-cell sequencing reveals the transcript profiles of single cells, giving the possibility to differentiate among cell populations that are not distinguishable by cell surface markers and morphology. This approach has revolutionized our ability to study the immune system and allowed us to break through the bottleneck of oncology studies. In a murine model, two TNBC cell lines (4T1 and EMT6) were observed [164]. Zhou et al. identified the upregulation of the *ETV6* gene in TNBC using single-cell RNA-seq [165]. These studies highlight that single-cell sequencing could help us to dissect the intratumoral heterogeneity and identify the critical genes of TNBC. This novel methodology represents a clinically relevant tool to map specific cellular populations spatially and temporally through disease progression and along treatment.

## 5. Comparative Oncology

### 5.1. Epidemiology and Molecular Characteristics

**Figure 5:**
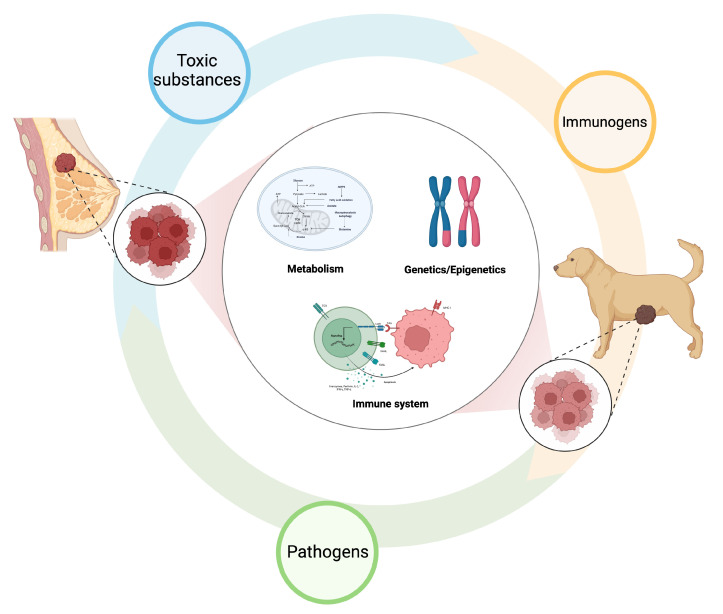
Comparative oncology in humans and dogs. Humans and dogs share similar living environments. Both these two species are exposed to similar toxic substances, immunogens, and pathogens. Because of these external stimuli, human breast cancers and canine mammary gland tumors develop from similar genetic/epigenetic alterations and metabolic changes. Furthermore, dogs are immunocompetent individuals, and the tumors share high similarity in tumor microenvironments with human counterparts.

Historical data suggest that almost half of CMTs are malignant [14,15,16]. Unfortunately, an increasing tendency toward malignant tumors has been observed over the years [17], especially in countries/regions where ovariectomy is not routinely performed. In female dogs and women, mammary cancer is the most frequently diagnosed malignancy and the leading cause of cancer-related death in women [12,166]. Furthermore, CMTs and HBCs share several similar characteristics in various aspects, such as hormonal receptors, metastatic patterns, and the roles of carcinogens, that impact the onset of these diseases [167]. Many pathological, molecular, and phenotypic similarities also exist between HBC and CMT [168,169]. Comparative genetic expression profiles revealed that cell cycle activation, WNT-β-Catenine signaling, PI3K/AKT, and ERK signaling were highly consistent in HBC and CMT. Furthermore, loss-of-function mutations in the tumor suppressors *CDKN2A*, *PTEN*, *CDH1*, and *TP53* were also observed in CMTs [170]. Because of these features, CMTs are a good spontaneous model for the study of HBC [171,172] (Figure 5).

Surgery is the treatment of choice in both HBC and CMT. Adjuvant therapies, especially hormone and targeted therapies, are only provided in HBC. One of the underlying reasons is that the question of whether dogs with CMTs might benefit from adjuvant therapies remains unanswered [173,174]. These results suggest that the clinical relevance between treatment choices and these hormone receptors remains poorly reported and still needs to be elucidated. Therefore, as previously noted for HBC, we summarized current investigations on hormone and targeted therapies for CMTs.

### 5.2. Hormone and Targeted Therapy in Canine Mammary Gland Tumors

#### 5.2.1. Hormone Therapy

In dogs, as in humans, hormones (estrogen and progesterone) manipulate the growth and development of mammary glands and participate in carcinogenesis [13]. The expression of ER and PR is correlated with better clinical outcomes in female dogs [175,176]. Decreased expression of ER and PR has been documented in canine malignant mammary tumors [177,178]. These findings reinforce that ER and PR are ideal therapeutic targets in CMTs. However, the expression of PR/ER remains undetermined in canine CMTs. ER and/or PR are expressed in about 66% of all malignant MGT cases [178], which is highly consistent with humans. Conversely, one publication reported that nearly 70% of MGT samples were negatively stained for ER or PR [179]. In human medicine, ER+ breast cancer is the most common subgroup (above 70%) [180].

Tamoxifen is widely utilized in the treatment of human breast cancer (HBC). However, in dogs, severe adverse effects (vulvar edema, vaginal purulent discharge, and pyometra) are repeatedly reported, and they outweigh the possible benefits of this hormone therapy [181,182]. Other anti-estrogen therapies, such as indole-3-carbinol (an estrogen receptor antagonist), have been studied for the treatment of CMTs. These studies were in vitro [183,184] or murine studies [185], and further clinical trials assessing the efficacy and safety are needed. Furthermore, despite the effectiveness of AIs as hormone therapy, no clinical trials on the use of this strategy in dogs with CMTs have been reported. Therapeutic targeting of PRs has been well studied in HBC, but anti-progestin has recently been employed in veterinary oncology. Mifepristone and onapristone can decrease the viability of CMT cell lines [186]. Aglepristone, a PR antagonist, effectively diminished the expression of PR and the proliferation of CMTs. Furthermore, dogs that benefited from aglepristone had increased PFS and overall survival time [187]. In addition to that trial, several underdeveloped hormone therapies remain in veterinary medicine. However, severe side effects and limited clinical studies in dogs might restrict further explorations.

#### 5.2.2. Her2-Targeted Therapies

The targeted therapy with anti-Her2 agents in HBC is well established. In veterinary medicine, to clarify the application of trastuzumab that can be applicable in dogs, the sequences and epitopes of the Her family have been analyzed. The amino acid homology values of Her1 and Her2 are, respectively, 91% and 92% between canines and humans. Cetuximab (antibody against Her1) epitopes only differ by four amino acids in dogs, and the trastuzumab (antibody against Her2) binding sites were identical in humans and dogs in an in silico study. Furthermore, cetuximab or trastuzumab significantly inhibited CMT cell growth and triggered cell cycle arrest [188]. However, no clinical trials have been published on the use of Her2 inhibitors or “caninized” monoclonal antibodies in CMTs. One of the possible reasons is that the clinical benefits of Her2 amplifications and their association with Her2 overexpression are not straightforward in CMTs [189,190]. Her2 status has been investigated in numerous studies to emphasize the similarity between humans and dogs, but validated methods for canine-specific Her2 clinical relevance are still lacking [191]. These unique findings indicate that the incidence and the clinical significance of Her2 in MCTs need to be further elucidated. Therefore, the role of Her2 expression in CMT needs to be scrutinized to determine its values for diagnosis, therapeutics, and prognosis in dogs.

The use of hormone and Her2-targeted therapies in canines is still far from becoming routine. Despite the similarities between humans and dogs, CMTs show certain discrepancies that have perplexed researchers (Table 3). Furthermore, no consistent classification based on HRs exists, and whether dogs with MCTs might benefit from hormone/Her2-targeted therapies still needs to be elucidated in the future.

## 6. Conclusions and Future Prospectives

In conclusion, our exploration of clinical trials in hormone receptor-positive breast cancer (HR+ BC) revealed a transformative era marked by the integration of conventional hormone therapy with targeted medicines. The field of hormone therapies for breast cancer has witnessed significant advancements, particularly with the success of SERMs like tamoxifen and Ais, such as anastrozole, letrozole, and exemestane. However, several challenges should be addressed, mainly in the form of resistance mechanisms. Combinational therapy, such as the use of AIs following tamoxifen, has shown promising efficacies, offering clinical benefits and reducing recurrence risk. The success of inhibitors targeting specific pathways and molecules in clinical trials has introduced novel strategies for treating HR+ BCs. The diverse range of targeted therapies reflects a growing understanding of the molecular complexities of breast cancer. Combining these therapies, exploring novel agents, and addressing challenges related to resistance and side effects are critical for advancing breast cancer treatment and improving patient outcomes. However, in the context of canine mammary tumors (CMTs), hormone therapy for dogs has often resulted in severe adverse effects, overshadowing the potential therapeutic benefits. The utilization of Her2 or other targeted therapies in dogs is still in the developmental stage and has not become routine.

While dogs are considered excellent models for human oncology, there is substantial room for improvement in developing novel therapies in veterinary medicine. Clinical trials in dogs with CMTs are limited, with few demonstrating effective anti-tumor efficacy and manageable side effects. Notably, novel immunotherapies in dogs with CMTs have been reported gradually, but the low case numbers hinder the derivation of conclusive and extrapolative results. To address these gaps, future research directions are recommended. First, conducting well-planned, large-scale, prospective randomized clinical trials in dogs with CMTs might obtain robust and conclusive results. Through these trials, a more comprehensive understanding of the efficacy and safety of hormone therapy, targeted therapies, and novel immunotherapies can be proposed. Second, dogs with spontaneous CMTs are ideal natural models for studying HR+ BC due to their similar physiological behaviors shared with humans. Immunocompetent dogs can mimic the complete immune system against cancer cells, reflecting the current state of cancer research and offering insights into trends in immune system activation. Last, comparative studies should be performed. Dogs with CMTs can provide more relevant and translatable insights into patients with HR+ BC compared to rodent models. Therefore, dogs are valuable bridges between preclinical research and clinical trials in humans. Through these concerted efforts, future research in veterinary medicine can significantly contribute to the development of effective and safe therapies for both dogs with CMTs and humans with BC. This integrated approach holds promise for advancing our understanding of cancer treatment, especially in the context of hormone and targeted therapies, and shaping the landscape of clinical oncology for both species.

## Figures and Tables

**Table 1:** Previous clinical trials reporting hormone therapies against breast cancers. BC, breast cancer; HR+, hormone receptor positive; PFS, progress-free survival; AEs, adverse effects.

Drugs	Indication	Efficacy	References or Accession Number
Tamoxifen	Late or recurrent carcinoma of the breast	22% of patients responded to treatment	[23]
	Hormone receptor-positive BC	Increased PFS	[25]
	Early breast cancer	Increased PFS and decreased death	[26]
Anastrozole	Patients with invasive operable breast cancer who had completed primary therapy	Anastrozole is an effective and well-tolerated with fewer AEs than tamoxifen.	[32,33]
Letrozole	HR+ BC (phase 3 trial)	Letrozole reduced the risk of recurrent diseases as compared with tamoxifen.	NCT00004205

**Table 2:** Previous clinical trials using small molecular inhibitors in treating breast cancers.

Medical Intervention		Phase	Indication	Efficacy	Reference or Accession Number
PI3K inhibitors	Buparlisib	3	HR+/Her2− BC	Buparlisib plus fulvestrant: well-tolerated. Serious AEs: increased alanine aminotransferase and aspartate aminotransferase	NCT01610284 (BELLE-2)
		3	HR+/Her2− BC	Buparlisib plus fulvestrant: increased PFS Serious AEs: increased alanine aminotransferase, increased aspartate aminotransferase, hyperglycemia, hypertension	NCT01633060 (BELLE-3)
		2/3	Her2− BC	No difference in PFS (compared to paclitaxel)	[51] (BELLE-4)
	Pictilisib	2	HR+/Her2− BC	No difference in PFS (compared to fulvestrant)	NCT01437566
		2	HR+/Her2− BC	No significant benefit from adding pictilisib to paclitaxel	NCT01740336 (PEGGY)
	Alpelisib	1	HR+/Her2− BC	Well-tolerated	NCT01219699
		1b	ER+/HT-resistant BC	Well-tolerated	NCT01219699
		3	HR+/Her2− BC	Alpelisib-fulvestrant prolonged PFS	NCT02437318
AKT inhibitors	Capivasertib	2	ER+/Her2− BC	Increased PFS	NCT01992952 (FAKTION)
		2	Metastatic TNBC	Increased PFS and OST	[52]
mTOR inhibitors	Everolimus	2	ER+ BC	Everolimus significantly increased letrozole efficacy.	NCT00107016
		2	HT-resistant BC	Everolimus plus tamoxifen increased clinical benefit rate, PFS, and OST.	[53]
		3	HR+ BC	Everolimus plus an AI increased PFS.	NCT00863655
CDK4/6 inhibitors	Palbociclib	3	HR+/Her2− BC	Palbociclib plus letrozole increased PFS.	NCT01740427
		3	HR+/Her2− BC	Palbociclib plus fulvestrant increased PFS.	NCT01942135
	Ribociclib	3	HR+/Her2− BC	Ribociclib plus letrozole increased PFS but a higher rate of myelosuppression was found.	NCT01958021
		3	HR+/Her2− BC	Ribociclib plus fulvestrant increased PST and OST	NCT02422615
	Abemeciclib	2	HR+/Her2− BC	Well-tolerated	[54] (MONARCH 1)
		3	HR+/Her2− BC	Improved PFS, ORR, and a tolerable safety profile were found.	NCT02107703 (MONARCH 2)
HDAC inhibitors	Tucidinostat	3	HR+ BC	Tucidinostat plus exemestane improved PFS; however, grade 3–4 hematological AEs were more common.	NCT02482753
PARP inhibitors	Olaparib	3	Her2− BC	Well-tolerated and increased PFS were found, but there was no statistically significant improvement in OST.	NCT02000622
	Talazoparib	3	Advanced BC with *BRCA1/2* mutation	Talazoparib increased PFS; however, no significant increased OST was found.	NCT01945775

**Table 3:** Comparative table of currently used anti-BC or anti-CMT therapies in humans and dogs.

		Human	Dogs
Hormone therapy	Tamoxifen	FDA-approved	Serious AEs in dogs
	AIs	FDA-approved	-
	Aglepristone	-	Increased PFS and OST in dogs
	Mifepristone	-	CMT cell lines
	Onapristone	-	CMT cell lines
Targeted therapy	Her-2 mAbs	FDA-approved	-
	Trastuzumab biosimilars	FDA-approved	-
	Tyrosine kinase inhibitors	FDA-approved	-
	PI3K/AKT/mTOR inhibitors	FDA-approved	-
	HDAC inhibitors	FDA-approved	-
	PARP inhibitors	FDA-approved	-

## Data Availability

Not applicable.
